# Barbiturates and pyrazolopyridines for the treatment of postpartum depression—repurposing of two drug classes

**DOI:** 10.3389/fphar.2023.1139889

**Published:** 2023-02-23

**Authors:** Alexander B. Horwitz, Robert T. Rubin

**Affiliations:** ^1^ Department of Graduate Medical Education, Community Memorial Healthcare, Ventura, CA, United States; ^2^ Department of Psychiatry and Biobehavioral Sciences, David Geffen School of Medicine at UCLA, Los Angeles, CA, United States

**Keywords:** postpartum depression, pyrazolopyridines, cartazolate, tracazolate, barbiturates, phenobarbital, neurosteroids, GABAA receptor

## Abstract

Zulresso (brexanolone) is an aqueous formulation of the neurosteroid, allopregnanolone, and the only FDA-approved medication for the treatment of postpartum depression (PPD). While brexanolone is effective for the treatment of PPD, lengthy infusion time and high cost can be prohibitive. Failure of GABA_A_ receptors to adapt to fluctuating neurosteroid levels is considered to predispose women to mood disorders in the postpartum period. Brexanolone is thought to act *via* stimulation of δ subunit-containing GABA_A_ receptors, which are extrasynaptic and localized to particular brain regions. Neurosteroid stimulation of δ subunit-containing GABA_A_ receptors leads to sustained inhibition (hyperpolarization) of GABAergic neurons, which makes δ subunit-containing GABA_A_ receptors a potentially important pharmacologic target. Barbiturates and pyrazolopyridines are potent stimulators of δ subunit-containing GABA_A_ receptors and therefore potentially cost-effective treatments for PPD. Barbiturates are often not prescribed, owing to risk of dependence and respiratory depression. The pyrazolopyridines were tested several decades ago for anxiety and depression but never developed commercially. Herein we use the FDA-approved dosing schedule of brexanolone and GABA_A_ receptor binding data from various animal models to examine the safety, efficacy, and potential clinical utility of barbiturates and pyrazolopyridines for the treatment of PPD. We suggest consideration of repurposing barbiturates and pyrazolopyridines as safe and readily available treatment alternatives for PPD.

## Introduction

PPD is an often underdiagnosed and debilitating mood disorder. The Diagnostic and Statistical Manual of Mental Disorders, 5th Edition, defines PPD as a major depressive disorder that occurs during pregnancy or within 4 weeks of birth (American Psychiatric Association, 2013). Women may be at increased risk of depression for as long as a year after birth (Gaynes et al., 2005), and PPD can last for up to 3 years after birth (Putnick et al., 2020).

PPD is a serious psychiatric complication of pregnancy and affects 10–25 percent of women who give birth (Halbreich and Karkun, 2006; Norhayati et al., 2015). It can have profound consequences, including incapacitating depressive symptoms in the mother and loss of interest and neglect of her newborn (Slomian et al., 2019). Instances of both postpartum suicide and infanticide by the depressed mother have been reported (Hatters-Friedman and Sorrentino, 2012). Antidepressants often are used for treatment, but some women are non-responsive, so that more effective treatments are very much needed.

## Mechanism of postpartum depression

Failure of GABA_A_ receptors to adapt to fluctuating neurosteroid levels is considered to predispose women to mood disorders in the postpartum period (Maguire and Mody, 2008). Pregnancy causes a rise in progesterone, which subsequently increases the progesterone metabolite and neurosteroid, allopregnanolone. Increased levels of allopregnanolone cause a compensatory decrease in GABA_A_ receptor number. Following parturition, progesterone and allopregnanolone levels decrease, leading to an increase in GABA_A_ receptor number. It is postulated that the inability of GABA_A_ receptors to increase in response to decreasing levels of allopregnanolone leads to PPD (Maguire and Mody, 2008; Osborne et al., 2017).

The GABA_A_ receptor is a heteropentameric ligand-gated ion channel. Up to 19 different subunits (α1–6, β1–3, γ1–3, δ, ε, θ, π, and ρ1–3) of the GABA_A_ receptor have been identified. The most common GABA_A_ receptor configuration is two α′s, two β′s, and a γ (α_2_β_2_γ). GABA (γ-aminobutyric acid), the major inhibitory neurotransmitter, is the endogenous ligand of the GABA_A_ receptor. When GABA binds to the GABA_A_ receptor, a chloride channel opens and allows selective permeability of the chloride ion, which leads to cell hyperpolarization. There are two different types of inhibition caused by GABA_A_ receptors: phasic and tonic. Phasic inhibition is transient and mediated by synaptic receptors, while tonic inhibition is more sustained and mediated by extrasynaptic receptors.

It is postulated that allopregnanolone specifically exerts its effect through the GABA_A_ receptor δ subunit (Maguire and Mody, 2009). In women with PPD, fluctuating allopregnanolone levels following pregnancy lead to the inability of δ subunit-containing GABA receptors to adapt. *δ* subunits are found exclusively at extrasynaptic sites and are therefore responsible primarily for tonic inhibition. Within the brain, *δ* subunits are primarily expressed in cerebellar granule cells and the dentate molecular layer but also are found in the thalamus, subiculum, cerebral cortex, and striatum (Peng et al., 2002; Uusi-Oukari and Korpi, 2010). Large changes in GABA concentrations lead only to slight changes in the current of *δ* subunit-containing GABA_A_ receptors. Although benzodiazepines are allosteric modulators of the GABA_A_ receptor, they have negligible activity against GABA_A_ receptors that contain a *δ* subunit. *δ* subunit-containing GABA_A_ receptors thus represent a unique pharmacologic target.

## Targeted treatment of postpartum depression

Zulresso (brexanolone) is an aqueous formulation of the neurosteroid, allopregnanolone, and, at the time of this writing, the only FDA-approved medication for the treatment of PPD. While brexanolone is an effective treatment, lengthy intravenous infusion (60 h) is required, at a very high cost ($34,000) (Edinoff et al., 2021). Given the time and expense required for brexanolone infusion, it is important to consider the therapeutic potential of alternative classes of compounds that are modulators of δ subunit-containing GABA_A_ receptors. Both barbiturates and pyrazolopyridines are potent modulators of δ subunit-containing GABA_A_ receptors and therefore are potential candidates for the treatment of PPD (Adkins et al., 2001; Zheleznova et al., 2008).

Barbiturates have largely been replaced by benzodiazepines in clinical practice, owing to the negative side effect profile of barbiturates, which includes increased risk for addiction and dependence and greater propensity for overdose and death from respiratory depression. The half-life of phenobarbital is approximately 70 h and theoretically would only require a few doses compared to a 60-h infusion of brexanolone, which would mitigate the chance of abuse and overdose. Phenobarbital also can be given as an IV infusion and possesses linear kinetics. Compared to brexanolone, barbiturates would be advantageous given their low cost.

For women with PPD, it has been hypothesized that returning to third trimester levels of allopregnanolone would ameliorate depressive symptoms (Zulresso brexanolone, 2022). Dosing of brexanolone is based on achieving third trimester allopregnanolone levels, uptitrating slowly to prevent excessive sedation and tapering slowly to avoid withdrawal. The 60-h infusion thus is as follows: 4 h at 30 μg/kg/hour, 20 h at 60 μg/kg/hour, 28 h at 90 μg/kg/hour, 4 h at 60 μg/kg/hour, and 4 h at 30 μg/kg/hour [Zulresso (brexanolone) NDA].

## Discussion: Barbiturates

Regarding the relative dosing of brexanolone to that of a barbiturate, Morrow et al. (1989) compared allopregnanolone to pentobarbital in the potentiation of muscimol-stimulated radioactive chloride uptake by GABA_A_ receptors in rat cortical synaptoneurosomes. The EC50 (half-effective concentration of the maximal response; Emax) of allopregnanolone was 110 nM, and the EC50 of pentobarbital was 20,000 nM. Allopregnanolone thus is 182 times more potent than pentobarbital in producing an equivalent EC50 in that test paradigm.

As an example, an 80 kg female having a 60-h brexanolone infusion according to the above recommended schedule would receive 4 h at 2,400 μg/h, 20 h at 4,800 μg/h, 28 h at 7,200 μg/h, 4 h at 4,800 μg/h, and 4 h at 2,400 μg/h, equating to a total of 336,000 μg allopregnanolone. A corresponding amount of pentobarbital would be (336,000 μg) (182) = 61,152,000 μg (61.152 g). The lethal dose of pentobarbital is approximately 10 g; thus, infusing pentobarbital in an amount approximating the effect of allopregnanolone on GABA_A_ receptors would require over six times its lethal dose.

Despite the calculation above, there is evidence to suggest that phenobarbital might have efficacy in depression and possibly PPD. Lambert el al. (2022) compared the EEG in mice exposed to non-sedative doses of allopregnanolone, ketamine, pentobarbital and diazepam, given in comparable doses: allopregnanolone 5 mg/kg, pentobarbital 15 mg/kg, and diazepam 1 mg/kg. It was hypothesized that the EEG signature of the rapidly acting antidepressant, allopregnanolone, would differ from those of diazepam and pentobarbital. The EEG of mice given allopregnanolone, however, was indistinguishable from that following a comparable dose of pentobarbital, and both were distinct from the EEG following diazepam. These results were surprising, given the purported selectivity of allopregnanolone for δ subunit-containing GABA_A_ receptors. The authors noted, “we are tempted to speculate that barbiturates in fact may share desirable psychotropic properties with AlloP [allopreganolone] at sub-sedative doses if EEG signatures identify those desirable properties” (Lambert el al., 2022, p. 376).

In our above example, what if the ratio of pentobarbital to allopreganolone in Lambert et al. (2022) were to be used instead of the conversion factor obtained from Morrow et al. (1990)? If 336,000 μg allopregnanolone is multiplied by a factor of three (15 mg/kg pentobarbital vs. 5 mg/kg allopregnanolone), 1,008,000 μg pentobarbital (1.008 g) is required to produce a similar effect, which is only about 1/10 the lethal dose.

## Pyrazolopyridines

The second class of compounds considered is the pyrazolopyridines (cartazolate, tracazolate, etazolate, etc.). Pyrazolopyridines were studied several decades ago for depression and anxiety but were not used in clinical practice or studied in the treatment of PPD. Pyrazolopyridines have similar effects on the GABA_A_ receptor as the barbiturates and, to different degrees, are adenosine A1 and A2 receptor antagonists and phosphodiesterase inhibitors as well (Patel et al., 1985; Ito et al., 1999).

Cartazolate was shown to have non-sedative anxiolytic and antidepressant properties in humans but was never developed commercially. In a small, open-label study (Sakalis et al., 1974), cartazolate, up to 200 mg per day, significantly reduced moderate-to-severe anxiety in 8/10 psychiatric inpatients. No side effects were reported. Similarly, in a small, open-label study (Sathananthan et al., 1976), 10 patients with severe psychotic depression were treated with cartazolate, up to 200 mg per day. Four of the 10 patients improved significantly. Of the six patients who did not respond to cartazolate, none responded to imipramine, a tricyclic antidepressant.

Tracazolate has anxiolytic, anticonvulsant, sedative, and muscle-relaxant effects. It has been used only in research. Tracazolate enhances the current amplitude of α_1_β_2_δ GABA_A_ receptors in the presence of saturating GABA concentrations more than does tetrahydrodeoxycorticosterone (THDOC)—a 23-fold vs. 3.4-fold increase. THDOC, like allopregnanolone, is a progesterone metabolite that can induce opening of the GABA_A_ receptor channel at nanomolar concentrations *in vitro*.

At present, there are no data which directly compare allopregnanolone to any pyrazolopyridine in terms of activity at *δ* subunit-containing GABA_A_ receptors. Ideally, the relative potencies of allopregnanolone and the pyrazolopyridines could be directly compared to determine if therapeutic concentrations of pyrazolopyridines can sufficiently stimulate δ subunit-containing GABA_A_ receptors at non-lethal doses. Although there are no binding data to compare allopregnanolone to the pyrazolopyridines in terms of activity at δ subunit-containing GABA_A_ receptors, there are data comparing tracazolate to THDOC (Zheleznova et al., 2008), which, as mentioned above, is a neurosteroid capable of opening the GABA_A_ receptor channel at nanomolar concentrations *in vitro*. The data suggest that tracazolate should induce opening of the *δ* subunit-containing GABA_A_ receptor channel at nanomolar concentrations as well.

As noted above, an 80 kg female having a 60-h brexanolone infusion would receive a total of 336,000 μg allopregnanolone. As tracazolate is clearly more potent than THDOC in terms of the enhancement of current amplitude of *δ* subunit-containing GABA_A_ receptors, it can be assumed that tracazolate would have an EC50 value less than the EC50 value for THDOC (344 nM). Thus, allopregnanolone is 3.13 times more potent [calculated by dividing the EC50 of THDOC (344 nM) by the EC50 of allopregnanolone (110 nM)] than THDOC, and therefore no more than 3.13 times more potent than tracazolate. Conservatively assigning an EC50 value of 3.13 nM to tracazolate, an amount of tracazolate equivalent to the allopregnanolone 60-h infusion would be (336,000 μg) (3.13) = 1,051,680 μg or approximately 1.052 g. It must be remembered that this amount is given over 60 h or 2.5 days, equating to approximately 420 mg of tracazolate per day.

Although there are no data comparing cartazolate to allopregnanolone or THDOC, there are data comparing cartazolate and tracazolate for GABA_A_ receptor activity. Using 35S-TBPS, Wong et al. (1984) compared the 50% binding concentrations (IC50s) of cartazolate and tracazolate to the GABA_A_ receptor in the cerebral cortex of mice. The IC50 for cartazolate was 500 nM while that for tracazolate was 2,200 nM, indicating that cartazolate was over four times more potent in inhibiting 35S-TBPS binding to the GABA_A_ receptor. It can be hypothesized that in clinical use, cartazolate would be approximately four times more potent than tracazolate; thus, the dose required to achieve a specific effect on GABA_A_ receptors would be correspondingly less than that of tracazolate. If tracazolate, therefore, can safely achieve similar activity to allopregnanolone at δ subunit-containing GABA_A_ receptors, at lower doses, cartazolate should as well.

## Potential limitations

There are several limitations to the comparisons detailed above. One is considering only EC50 values (relative potency) while not considering the Emax. The Emax could be taken into consideration mathematically, which is beyond the scope of this article. Morrow et al. (1990) found that THDOC had the highest Emax (14.2 mmol/mg protein), followed by pentobarbital (12.9 mmol/mg protein), and then allopregnanolone (10.9 mmol/mg protein). Because the EC50 values for pentobarbital and THDOC are lower than that of allopregnanolone, in theory both pentobarbital and THDOC could produce a greater effect than allopregnanolone. It also can be hypothesized that cartazolate and tracazolate could produce Emax’s higher than that of THDOC. The implications are that the high concentrations of pentobarbital, THDOC, cartazolate, and tracazolate required to produce a maximal effect could potentially be lethal in the clinical setting. This appears unlikely, however, given the calculations detailed above and the demonstrated efficacy of these compounds in the treatment of depression and anxiety (Sakalis et al., 1974; Sathananthan et al., 1976).

Another limitation is that the models discussed involve *in vitro* and animal studies: Morrow et al. (1990) examined potentiation of muscimol-stimulated radioactive chloride uptake by GABA_A_ receptors in rat cortical synaptoneurosomes; Zheleznova et al. (2008) examined GABA_A_ activated currents in α_1_β_2_δ GABA_A_ receptors in *Xenopus laevis* oocytes; and Wong et al. (1984) examined displacement of 35S-TBPS binding to the GABA_A_ receptor in the cerebral cortex of mice. The subunit composition of the GABA receptors in the Morrow et al. (1990) and Wong et al. (1984) studies was not specified and cannot be assumed to have contained the δ subunit, making comparisons between the two models difficult.

## Summary

We propose repurposing pyrazolopyridines, which have shown clinical efficacy in the treatment of depression and anxiety but which never have been developed commercially, as well as barbiturates, for the treatment of postpartum depression (PPD). Regarding the barbiturates, our calculations suggest that the dosage of pentobarbital needed to approximate the effect of the FDA-approved dosage schedule of allopregnanolone would be lethal in humans, although a recent EEG study (Lambert et al., 2022) suggests that pentobarbital potentially has an effect on neurocircuitry analogous to that of allopregnanolone. Of importance, pyrazolopyridines are potentially more potent stimulators of *δ* subunit-containing GABA_A_ receptors than are barbiturates. Small, open-label clinical studies indicate that cartazolate is both safe and effective for the treatment of depression and anxiety. Our calculations suggest that both cartazolate and tracazolate can be safely and effectively used to treat PPD in humans. The pyrazolopyridines, and possibly the barbiturates, offer a potential treatment for PPD that would be available in oral form, relatively safe, and not nearly so costly as allopregnanolone.

## Data Availability

The original contributions presented in the study are included in the article/Supplementary Material, further inquiries can be directed to the corresponding authors.
